# Utilization of magnification devices in Vietnam’s dental practice and education: an online survey

**DOI:** 10.1186/s12903-025-06306-6

**Published:** 2025-06-06

**Authors:** Minh-Hieu Nguyen Tran, Anh Huynh Bui, Lan-Anh Hoang Le, Thuy-Trang Thi Huynh, Phuong-Doan Phan, Thuan-Loc Tran, Nguyen-Tra-Mi Le, Thu-Tra Nguyen, Hoang-Vinh Le, Dai-Phong Lam, Quoc-Viet Lam, Tran-Lan-Khue Pham, An-Tran Pham, Van-Khoa Pham

**Affiliations:** 1 Department of Operative Dentistry and Endodontics, Faculty of Dentistry, University of Medicine and Pharmacy, Ho Chi Minh City, Viet Nam; 2 Department of Oral Radiology, Faculty of Dentistry, University of Medicine and Pharmacy, Ho Chi Minh City, Viet Nam; 3 Faculty of Dentistry, University of Medicine and Pharmacy, Ho Chi Minh City, Viet Nam

**Keywords:** Dental operating microscope, Dental loupe, Dental education, 3D Dental operating microscope, Ergonomics, Endodontics

## Abstract

**Background:**

Advancements in dental technology have enhanced diagnostic and treatment tools, with magnification devices improving clinical precision and ergonomics. Dental loupes, conventional dental operating microscopes (DOMs), and three-dimensional digital operating microscopes (3D-DOMs) help overcome visual limitations in intricate procedures. Although widely used globally, adoption in Vietnam remains underreported. This study aimed to assess magnification device utilization in Vietnamese dental practice and education, with a pioneering focus on 3D-DOMs.

**Methods:**

An online, questionnaire-based observational survey was conducted from October to December 2024. A total of 108 dentists responded, along with 11 dental education institutions and 5 hospitals (2 specialized, 3 general). The 28-item questionnaire, validated by a subject expert (CVI-S = 0.9), was distributed via email and messaging platforms. Google login ensured one response per participant. Data were analyzed using JASP (version 0.19.3) with descriptive statistics and Chi-square tests (*p* < 0.05).

**Results:**

Awareness was high (96.4%), while actual usage was moderate (62.96%). Usage varied by setting: specialized hospitals (100%) and education institutions (80%) showed higher adoption than general hospitals (33.33%). Dental loupes were most common (55.56%), followed by DOMs (25.93%) and 3D-DOMs (5.56%). Future usage intentions were high, particularly for loupes (81.48%). Endodontics was perceived as the specialty benefiting most. Despite cost being the primary barrier, satisfaction was high. Formal training significantly improved self-rated knowledge and satisfaction.

**Conclusions:**

Despite cost and ergonomic barriers, magnification devices—especially 3D-DOMs—show strong potential. With improved training and resource allocation, they could significantly enhance diagnostic precision and clinical outcomes in Vietnamese dentistry.

**Supplementary Information:**

The online version contains supplementary material available at 10.1186/s12903-025-06306-6.

## Introduction

The evolution of dental technology has led to significant advancements in diagnostic and treatment tools, among which magnification devices play a crucial role in enhancing clinical precision and efficiency [1]. The resolving power of the human eye is inherently limited, making it difficult for clinicians to achieve optimal accuracy in intricate dental procedures. To address this challenge, various optical aids have been introduced, revolutionizing both dental practice and education [2].

Magnification devices in dentistry primarily include dental loupes, dental operating microscopes (DOM), and three-dimensional digital operating microscopes (3D DOMs). Loupes have been widely used due to their accessibility and ability to enhance visualization at a relatively low cost. The dental operating microscope (DOM) was first introduced by Apotheker in 1981, but did not gain widespread acceptance until ergonomic improvements were made in the late 1990s [3]. Since then, DOM has become a fundamental tool in endodontics, restorative dentistry, periodontics, and microsurgery, significantly improving procedural outcomes. More recently, 3D DOM technology has emerged, offering a high-definition digitalized view of the operative field with freedom of movements, enhanced depth of field and enabling real-time magnification without requiring direct visualization through oculars. These technological advancements have contributed to both higher treatment precision and improved ergonomic conditions for clinicians [4].

The integration of magnification devices into clinical practice and dental education has provided multiple benefits. Enhanced visualization enables more precise diagnosis, minimally invasive procedures, and improved treatment outcomes, particularly in complex procedures such as endodontic therapy, microsurgical interventions, esthetic restorations [5–8]. Pediatric dentistry and bleaching might also benefit from magnification devices [9–11]. For example, magnified vision enables more precise detection and thorough cleaning of root canals through conservative access cavities, which may enhance the long-term retention and fracture resistance of treated teeth [12]. Additionally, the use of microscopes in targeted endodontic microsurgery helps minimize the risk of damaging vital anatomical structures by providing superior magnification, illumination, and visualization compared to conventional methods [13]. In esthetic restorations, microscopes facilitate precise control over minimally invasive tooth preparation, allowing for accuracy up to 0.1 mm [14]. In dental education, magnification tools support skill development by allowing students and trainees to observe fine details, maintain proper ergonomic postures, and refine clinical techniques under enhanced visualization. Magnification tools have been shown to improve operating posture compared to direct vision, with dental operating microscopes (DOMs) offering greater ergonomic benefits than loupes [14, 15].

In countries like the United Kingdom and the United States, studies have reported a growing trend in the utilization of magnification devices, particularly dental operating microscopes (DOMs) [16, 17]. However, DOMs were not widely used in some other countries like Turkey, Romania or India [18–20]. While global studies have examined the use of magnification tools, data from Vietnam remain limited. Despite their significant benefits, drawbacks such as high cost and a steep learning curve have influenced dentists’ decisions regarding their use [1]. In Vietnam, economic constraints, inconsistent institutional support, and variable training exposure may contribute to a uniquely different utilization pattern. These factors highlight the need for a localized investigation into adoption, challenges, and accessibility across various user groups.

This study aimed to assess the current state of magnification device adoption in Vietnam from three key perspectives: practicing dentists, heads of major hospitals, and leaders of dental education institutions. By evaluating awareness levels, utilization trends, benefits, and barriers, this research sought to provide insights into how magnification technology can be more effectively integrated into various aspects of Vietnamese dentistry. otably, this study also pioneered the investigation of 3D-DOM in the Vietnamese context—a technology that has scarcely been addressed in previous literature—thereby providing essential baseline data on its usage, benefits, and challenges in modern dental practice.

## Materials and methods

### Study design

An online observational, qualitative, questionnaire-based survey, approved by the Faculty Review Board, was conducted from October to December 2024.

### Participants and questionnaire

A letter and questionnaire regarding the usage of magnification devices (including loupes, DOM and 3D DOM) were distributed to dental education institutions, major hospitals specializing in dentistry or housing a dental department, and practicing dentists across Vietnam. Although all dental education institutions were invited to participate, individual participants were recruited through a convenience sampling strategy based on accessibility and availability. To enhance representativeness and minimize selection bias, the sample included individuals from diverse institutional, professional, and socio-demographic backgrounds across different regions of the country.

**Fig. 1:**
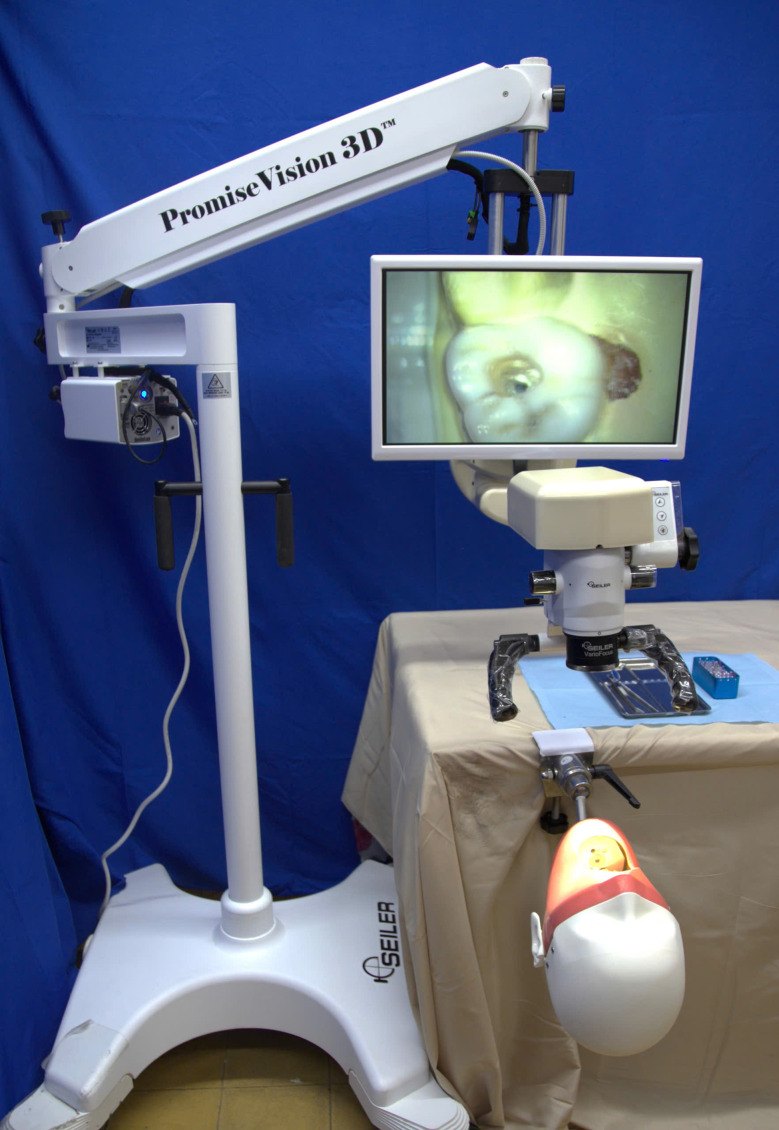
The 3D Dental operating microscope used at the University of Medicine and Pharmacy at Ho Chi Minh City

The questionnaire consisted of 28 close-ended questions (excluding demographic items) and was developed by a multidisciplinary research team. Content and construct validity were evaluated by a subject expert to ensure that each question was relevant, clear, and aligned with the study’s objectives. Following expert feedback and necessary revisions, a pilot test was conducted with 10 dentists from the Faculty of Dentistry, University of Medicine and Pharmacy, Ho Chi Minh City. Responses were analyzed using the Content Validity Index Score (CVI-S), resulting in a CVI-S of 0.9, indicating acceptable validity (See Fig. 1).

The finalized questionnaire was distributed both in person and via online platforms, including email and messaging services. To ensure data integrity and prevent duplicate responses, participants were informed about the single-submission nature of the survey and required to log in using their Google accounts, which restricted submissions to one per account. Once submitted, responses could not be edited during the data collection period.

Inclusion criteria encompassed dentists currently involved in academic or clinical practice in either public or private institutions, hospitals, or clinics. Exclusion criteria included retired dentists, those who had left the dental profession for more than six months, individuals who did not consent to participate, and those who submitted incomplete responses.

### Statistical analysis

Data from the Google Forms were analyzed using JASP, version 0.19.3 (JASP Team, University of Amsterdam, Amsterdam, The Netherlands). Simple descriptive statistics were employed to summarize the characteristics of the variables, presenting categorical data as numbers and percentages. To assess relationships between categorical variables, the Chi-square test was utilized, with a significance level set at *P* < 0.05.

## Results

A total of 108 responses were collected from dentists, 11 dental education institutions, and 5 hospitals (including 2 specialized dental hospitals and 3 general hospitals). The socio-demographic characteristics of the respondents are presented in Table 1.

**Table 1:** Socio-demographic data of the participants

Demographics		Have you ever used any magnification devices?		p
		Yes (%)	No (%)	
**Gender**	Female	34 (65.38%)	18 (34.62%)	0.762
	Male	34 (60.71%)	22 (39.29%)	
**Age**	< 30 years	24 (53.33%)	21 (46.67%)	0.0487
	30–35 years	20 (76.92%)	6 (23.08%)	
	41–45 years	15 (68.18%)	7 (31.82%)	
	36–40 years	6 (66.67%)	3 (33.33%)	
	46–50 years	0 (0.00%)	3 (100.00%)	
	> 50 years	3 (100.00%)	0 (0.00%)	
**Academic qualifications**	Dentist	59 (93.65%)	4 (6.35%)	< 0.001
	Specialist level I	14 (58.33%)	10 (41.67%)	
	Master of Science	7 (100.00%)	0 (0.00%)	
	Master of Science, Specialist level I, Residency	8 (100.00%)	0 (0.00%)	
	Master of Science, Specialist level II	4 (100.00%)	0 (0.00%)	
	PhD	2 (100.00%)	0 (0.00%)	
**Workplace**	Dental education institution	18 (75.00%)	6 (25.00%)	0.6251
	Specialized dental hospital	4 (57.14%)	3 (42.86%)	
	General hospital	18 (60.00%)	12 (40.00%)	
	Private clinic	46 (68.66%)	21 (31.34%)	

Among the participants, 96.4% were aware of magnification devices, yet only 62.96% had used them. Specialized dental hospitals (100%) and education institutions (80%) reported higher usage compared to general hospitals (33.33%). Educational institutions primarily used devices for teaching and research, whereas hospitals emphasized clinical applications (Tables 2 and 3). Educational institutions showed broader adoption of all three types of devices, especially loupes and DOM, while general hospitals had limited or no usage of DOM and 3D DOM. All dentists with a master’s degree or higher had used at least one type, whereas usage was lower among those with only dentist or specialist level I degrees (*p* < 0.05). Dentists aged 30–45 were the most frequent users compared to younger or older groups (*p* < 0.05). Guidance from experts (universities or suppliers) was linked to better self-rated understanding compared to self-study or peer learning (*p* < 0.05). Usage varied across specialties, with endodontics reporting the highest benefit from magnification devices, especially DOM and 3D DOM, due to the precision required in root canal treatments. Other specialties, such as orthodontics and oral surgery, showed lower utilization, likely because their procedures rely more on broader visualizations rather than fine details. Usage frequency (rated 3–5 on a 5-point scale) was 51.67% for loupes, 46.43% for DOM, and 50% for 3D DOM. Cost was the main barrier for dentists, institutions and hospitals despite widespread recognition of benefits such as enhanced visualization and improved ergonomics. Most users expressed satisfaction (96.67% for loupes, 89.29% for DOM, 100% for 3D DOM), as reflected by ratings of 3–5 on a 5-point scale. Additionally, 95.37% of surveyed dentists intended to use magnification devices in the future (See Tables 4 and 5).

**Table 2:** Descriptive analysis of participants’ responses regarding their usage status, purpose of use, perceived necessity, usage intentions, and satisfaction with magnification devices

		Loupes	DOM	3D DOM
**Usage status**	Dentists	60 (55.56%)	28 (25.93%)	6 (5.56%)
	Education institutions	8 (72.73%)	4 (45.45%)	1 (18.18%)
	Specialized dental hospital	2 (100.00%)	2 (100.00%)	1 (50.00%)
	General hospital	1 (33.33%)	0 (0.00%)	0 (0.00%)
**Dentists’ purposes of use**	Teaching	2 (2.15%)	3 (6.12%)	2 (20.00%)
	Researching	3 (3.23%)	8 (16.33%)	2 (20.00%)
	Diagnosing	29 (31.18%)	12 (24.49%)	1 (10.00%)
	Treatment	59 (63.44%)	26 (53.06%)	5 (50.00%)
**Dentists’ Self-Rated Necessity of Use** (average of all specialties)	1 – Not necessary	9.4 (8.70%)	10.6 (9.81%)	11.0 (10.19%)
	2 – Slightly necessary	10.2 (9.44%)	15.6 (14.44%)	15.0 (13.89%)
	3 – Moderately necessary	30.9 (28.61%)	47.1 (43.61%)	53.8 (49.81%)
	4 – Highly necessary	39.8 (36.85%)	23.1 (21.39%)	20.9 (19.35%)
	5 – Essential	17.7 (16.39%)	11.6 (10.74%)	7.3 (6.76%)
**Dentists’ Self-Rated Utilization Satisfaction**	1 – Very dissatisfied	1 (1.67%)	3 (10.71%)	0 (0.00%)
	2 – Somewhat dissatisfied	1 (1.67%)	0 (0.00%)	0 (0.00%)
	3 – Neutral / Acceptable	13 (21.67%)	6 (21.43%)	2 (33.33%)
	4 – Satisfied	31 (51.67%)	12 (42.86%)	1 (16.67%)
	5 – Very satisfied	14 (23.33%)	7 (25.00%)	3 (50.00%)
**Dentists’ usage intentions**	Magnification devices	88 (81.48%)	72 (66.67%)	46 (42.59%)
	No intention to use any devices	5 (4.63%)		

**Table 3:** Purposes of use of magnification devices across educational institutions, specialized hospitals, and general hospitals

Purposes of use	Educational Institutions			Specialized Hospitals			General Hospital		
	Loupes	DOM	3D DOM	Loupes	DOM	3D DOM	Loupes	DOM	3D DOM
**Graduate teaching**	4 (21.05%)	4 (26.67%)	2 (25.00%)	0 (0.00%)	1 (16.67%)	0 (0.00%)	0 (0.00%)	0 (0.00%)	0 (0.00%)
**Post-graduate teaching**	2 (10.53%)	2 (13.33%)	2 (25.00%)	0 (0.00%)	1 (16.67%)	0 (0.00%)	0 (0.00%)	0 (0.00%)	0 (0.00%)
**Science researching**	5 (26.32%)	4 (26.67%)	2 (25.00%)	0 (0.00%)	2 (33.33%)	0 (0.00%)	0 (0.00%)	0 (0.00%)	0 (0.00%)
**Diagnosis & treatment**	8 (42.11%)	5 (33.33%)	2 (25.00%)	2 (100.00%)	2 (33.33%)	1 (100.00%)	1 (100.00%)	0 (0.00%)	0 (0.00%)

**Table 4:** Self-Rated knowledge levels for magnification devices (Loupes, DOM, and 3D-DOM) according to different learning methods

Magnification devices	Dentists’ self-rated knowledge	No knowledge	Self-study	Peer-learning	Suppliers’ experts	Universities’ experts	*p*
**Loupes**	1 - very little or no knowledge	9	1	1	0	0	< 0.001
	2 - basic knowledge	9	7	11	3	1	
	3 - moderate knowledge	1	22	24	9	10	
	4 - good knowledge	0	9	4	6	4	
	5 - expert knowledge	0	3	3	4	5	
**DOM**	1 - very little or no knowledge	30	1	3	0	1	< 0.001
	2 - basic knowledge	16	8	15	3	2	
	3 - moderate knowledge	1	7	16	3	10	
	4 - good knowledge	0	3	3	4	3	
	5 - expert knowledge	0	1	2	2	1	
**3D – DOM**	1 - very little or no knowledge	63	0	1	1	0	< 0.001
	2 - basic knowledge	14	6	7	1	3	
	3 - moderate knowledge	1	3	7	1	2	
	4 - good knowledge	0	0	2	0	0	
	5 - expert knowledge	0	0	1	0	1	

**Table 5:** Barriers and benefits of magnification devices: overall responses vs. Device users’ responses

Barriers	All responses			Magnification devices users’ responses		
	Loupes	DOM	3D DOM	Loupes	DOM	3D DOM
High cost	43 (22.16%)	95 (38.46%)	95 (43.38%)	21 (18.92%)	26 (27.08%)	6 (30.00%)
Heavy on the head	34 (17.53%)	5 (2.02%)	6 (2.74%)	19 (17.12%)	2 (2.08%)	1 (5.00%)
Eye strain	24 (12.37%)	16 (6.48%)	7 (3.20%)	12 (10.81%)	7 (7.29%)	0 (0.00%)
Uncomfortable posture	24 (12.37%)	14 (5.67%)	6 (2.74%)	13 (12.62%)	5 (6.67%)	0 (0.00%)
Difficult to operate	15 (7.73%)	21 (8.50%)	14 (6.39%)	7 (6.31%)	9 (9.38%)	1 (5.00%)
Blurred or double images	8 (4.12%)	8 (3.24%)	2 (0.91%)	0 (0.00%)	0 (0.00%)	0 (0.00%)
Difficult maintenance	9 (4.64%)	28 (11.34%)	31 (14.16%)	6 (5.41%)	8 (8.33%)	3 (15.00%)
Bulky design	7 (3.61%)	30 (12.15%)	25 (11.42%)	4 (3.60%)	10 (10.42%)	2 (10.00%)
Prolonged treatment time	5 (2.58%)	9 (3.64%)	9 (4.11%)	4 (3.60%)	4 (4.17%)	0 (0.00%)
Complicated to use	3 (1.55%)	19 (7.69%)	20 (9.13%)	1 (0.90%)	3 (3.13%)	0 (0.00%)
No significant limitations	22 (11.34%)	2 (0.81%)	4 (1.83%)	16 (14.41%)	1 (1.04%)	1 (5.00%)
**Benefits**						
Enhanced visualization	89 (28.16%)	83 (27.48%)	79 (26.69%)	50 (26.32%)	25 (29.76%)	6 (28.57%)
Precision in operation	71 (22.47%)	78 (25.83%)	78 (26.35%)	41 (21.58%)	19 (22.62%)	4 (19.05%)
Improved ergonomics	59 (18.67%)	59 (19.54%)	57 (19.26%)	35 (18.42%)	19 (22.62%)	5 (23.81%)
Treatment with small instruments	59 (18.67%)	72 (23.84%)	70 (23.65%)	39 (20.53%)	19 (22.62%)	4 (19.05%)
Reasonable cost	29 (9.18%)	1 (0.33%)	1 (0.34%)	18 (9.47%)	0 (0.00%)	0 (0.00%)
Infection control	9 (2.85%)	9 (2.98%)	11 (3.72%)	7 (3.68%)	2 (2.38%)	2 (9.52%)

## Discussion

This study provided a comprehensive evaluation of the utilization of magnification devices—including dental loupes, conventional dental operating microscopes (DOM), and the emerging 3D digital operating microscopes (3D-DOM)—in Vietnam’s dental practice and education. Our findings revealed current usage trends, satisfaction levels, and perceived challenges among clinicians and educators, while also underscoring the pioneering nature of surveying a new technological adjunct—3D-DOM—which had scarcely been addressed in previous literature.

### Innovative Contributions and Multi-Stakeholder Perspectives

A significant contribution of our study was its multi-stakeholder approach. Unlike previous surveys that typically focused solely on practicing dentists [11, 21]our research captured insights from practicing dentists, heads of major hospitals, and leaders of dental education institutions. This inclusive approach enriched our understanding of how magnification devices were integrated across different layers of dental care and training. Although awareness of magnification devices was high (96.4%), actual usage stood at 62.96%, with usage rates varying markedly by setting. For example, specialized dental hospitals and educational institutions reported higher usage compared to general hospitals—a variation that may have reflected differences in institutional priorities, resource allocation, and training infrastructure.

The effectiveness of self-administered surveys in educational settings is exemplified by Puleio et al. (2025), who used structured questionnaires to assess student experiences with Ni-Ti rotary systems [22]. Similarly, our study could benefit from incorporating well-designed questionnaires that evaluate participants’ experiences, focusing on key variables relevant to the research. Using Likert-scale questions and focused prompts enables efficient data collection and facilitates statistical analysis, providing structured feedback that can inform improvements in educational protocols and clinical practice.

### Comparative analysis of device types

When assessing future usage intentions, 81.48% of respondents expressed intent to continue or start using loupes, 66.67% for DOMs, and 42.59% for 3D-DOMs. Dental loupes, with a usage rate of 55.56%, remained the most commonly adopted tool across the board, likely due to their cost-effectiveness and ease of use. In contrast, conventional DOMs were used by 25.93% of respondents, and 3D-DOMs, though the least adopted at 5.56%, were associated with high satisfaction levels. Among those who used 3D-DOMs, 50% reported being “very satisfied,” suggesting that once adopted, the advanced features of 3D visualization and enhanced ergonomics had a significant positive impact on clinical practice.

### Pioneering the survey of 3D-DOM

Incorporating 3D-DOM into our survey represented a novel contribution. Our findings provided essential baseline data on its current usage, benefits, and challenges. Notably, 3D-DOMs have demonstrated greater efficacy than dynamically guided trephining with extractors in the retrieval of separated endodontic [23]. Although fewer participants used 3D-DOMs compared to loupes or conventional DOMs, their high satisfaction ratings indicate these devices may be particularly beneficial for procedures requiring high precision [24]. Enhanced digital imaging and the ability to operate without traditional ocular constraints could transform specialties where detailed visualization is critical, such as endodontics and microsurgery [4, 24].

### Demographic and educational influences on adoption

Our data revealed that the adoption of magnification devices was significantly influenced by demographic and educational factors. Dentists aged 30–45 were the most frequent users, likely due to a combination of clinical experience, financial capacity, recent exposure to modern training emphasizing magnification, and a higher likelihood of postgraduate education. At this career stage, they are more inclined to invest in long-term tools, particularly as they begin to experience musculoskeletal discomfort, further motivating the use of ergonomically beneficial devices. Additionally, all dentists with a master’s degree or higher reported using at least one magnification device, compared to lower usage among those with only basic qualifications. Training obtained through expert guidance—whether from universities or suppliers—resulted in a significantly higher level of self-rated knowledge and satisfaction compared to self-study or peer learning. This observation aligns with the findings of Iqbal et al., which suggest that magnification devices should be integrated into the official educational curriculum, enabling students to learn and practice properly from the outset [21]. Overall, these findings underscore the critical importance of structured, formal training programs in facilitating the adoption of advanced technologies in dental practice.

### Practical benefits and clinical applications

The survey results clearly highlighted the practical benefits of using magnification devices in clinical settings. Enhanced visualization emerged as the most frequently cited benefit, directly contributing to improved diagnostic accuracy and treatment precision. In endodontics and microsurgery—fields that demand high precision—the improved visualization and ergonomics provided by these devices led to significantly better clinical outcomes. In these specialties, even basic devices like loupes were deemed essential for locating root canals, while more advanced systems such as DOMs and 3D-DOMs further refined treatment quality. Moreover, the improved ergonomics of these advanced systems reduced physical strain on clinicians during long, intricate procedures, thereby supporting sustained performance and overall practitioner well-being [25].

Our survey examined the frequency with which dental professionals used magnification devices—dental loupes, conventional dental operating microscopes (DOMs), and 3D digital operating microscopes (3D-DOMs)—across a range of specialties on a scale of 1 (lowest) to 5 (highest). In general, dental loupes were reported as the most frequently used tool across most procedures. In contrast, 3D-DOMs, being a newer technology, showed a tendency toward lower frequency usage overall—with a significant portion of users reporting low-frequency ratings. Frequency data underscored the high dependence on magnification devices in endodontics. Only about 5% of endodontists rated loupes at the lowest frequency, compared to roughly 43% in diagnostic fields. Similarly, only around 3.6% of endodontists rated DOM usage as very low, versus over 70% in other specialties. For 3D-DOMs, only about 17% of endodontists rated usage at the lowest level, compared to 83% in other specialties—indicating a much higher, more consistent use in endodontic practice. Endodontists tended to rate 3D-DOM usage at moderate levels, with nearly 33% each giving ratings of 2 and 3. These trends suggest that for endodontic procedures—where precision is paramount—advanced visualization tools are employed more consistently. Similar results were observed in previous studies, which identified endodontics as the primary application for magnification devices among all dental specialties [19, 21, 26].

Focusing on specific endodontic tasks, our data showed that nearly half of the respondents used loupes (approximately 50%), while conventional DOMs and 3D-DOMs were employed at similar rates (around 43% each). For instrument retrieval—a procedure demanding exceptional clarity and depth perception—usage increased for 3D-DOMs (approximately 35.7%) compared to about 30% for both loupes and conventional DOMs. In managing ledges and perforations, the use of conventional DOMs was slightly higher than that of loupes, suggesting a preference for enhanced imaging in more challenging cases. Moreover, previous research by Kovács-Ivácson et al. reported that complex cases—such as hidden canals, calcified root canals that require penetration, or the removal of fractured instruments—are precisely the scenarios where dentists tend to utilize their DOMs [19]. Overall, these findings underscore that while loupes remain a fundamental tool in endodontic procedures, more advanced systems like DOMs and 3D-DOMs are increasingly adopted in cases where precision in small details is particularly critical.

Perceived necessity in endodontics was also high. Approximately 91% of respondents rated loupes as highly necessary or essential, while conventional DOMs received similarly robust endorsements. Although 3D-DOMs had a somewhat lower “essential” rating (about 25%), highlighting a strong overall endorsement despite their lower frequency of use. These findings underscore the critical role that advanced magnification devices play in improving treatment precision and patient outcomes in endodontics. Beyond endodontics, other specialties such as implantology, restorative dentistry, and oral surgery also showed high perceived necessity for magnification devices, though the exact ratings varied. The overall trend was clear: while basic devices like loupes remain indispensable across the board, advanced systems (DOMs and 3D-DOMs) are increasingly valued in areas where enhanced precision can directly impact treatment outcomes.

### Barriers to adoption and future directions

Despite the recognized benefits, several barriers hindered the widespread adoption of magnification devices, particularly the more advanced DOM and 3D-DOM systems. High cost was identified as the most significant barrier, with 38.46% of all responses citing cost-related issues for DOMs and 43.38% for 3D-DOMs. These financial constraints were especially pronounced in resource-limited settings, where general hospitals tended to rely solely on more cost-effective devices like loupes. Additionally, the advanced devices remain prohibitively expensive in many areas, particularly in developing countries like Vietnam, further limiting their widespread adoption [11, 27]. Furthermore, reported challenges such as device weight, eye strain, and maintenance issues were noted. Similar disadvantages have also been noted in previous studies [16, 28]. Interestingly, these challenges were less frequently mentioned by active users, suggesting that familiarity and practical experience can mitigate some concerns over time. In particular, DOM and 3D-DOM have a steep learning curve [1, 24, 29]. First-time users often encounter significant difficulties when operating these devices, which may exacerbate issues such as eye strain, poor posture, and longer treatment times before they master the technology and fully benefit from its advantages. This phenomenon may explain why uncomfortable posture was frequently reported as one of the most cited disadvantages by experienced, yet not fully proficient, users in our study. These barriers need to be addressed through policy initiatives, funding programs, and improvements in device design. Specifically, structured training on ergonomics and posture could help mitigate discomfort, while institutional subsidies, shared equipment models, or government-supported leasing programs could improve access to advanced magnification devices across Vietnam. Future research should focus on long-term, outcome-based evaluations to further substantiate the clinical benefits of advanced magnification devices, and technological improvements that addressed current barriers—particularly cost and ergonomic issues—remained critical.

### Implications for dental education and policy

The disparate purposes of use across educational institutions, specialized hospitals, and general hospitals highlighted the need for tailored approaches in both education and clinical practice. In educational institutions, magnification devices were used not only for clinical diagnosis and treatment but also extensively for graduate and post-graduate teaching as well as scientific research. This diversified use suggested that integrating magnification technology into the dental curriculum could enhance both theoretical and practical training, thereby preparing future dentists to adopt advanced technologies seamlessly. Emphasizing hands-on training and expert-led workshops appeared to bridge the gap between awareness and practical application, ultimately leading to more consistent and effective use in clinical practice.

In specialized hospitals, advanced magnification devices like DOMs and 3D-DOMs were used not only for diagnosis and treatment but also for teaching and research, highlighting their dual role in clinical care and academic advancement. In contrast, general hospitals primarily relied on loupes, likely due to budget constraints and a lower perceived need for advanced technologies in routine cases. Given their broader scope of use, specialized hospitals could serve as models for other institutions.

### Limitations

Although it successfully captured responses from a broad range of dental professionals and institutions across Vietnam, the sample size was not predetermined through a formal power analysis and participation was voluntary, leading to a possibility of selection bias. Additionally, as with most online surveys, response bias cannot be entirely excluded. To improve generalizability, future studies should consider using stratified random sampling methods and larger, systematically determined sample sizes.

## Conclusion

In summary, this study offers the first comprehensive look at magnification device use in Vietnam’s dental practice and education with multiple perspectives, notably including 3D-DOM. Despite barriers like cost and ergonomics, high satisfaction and strong future-use intent among early adopters highlight their transformative potential. Moreover, our results underscored that organized and formal training programs play a crucial role in promoting the proper integration of advanced technologies into dental practice. These findings support integrating magnification training into dental curricula and continuing education to build proficiency early and consistently.

Immediate steps—such as institutional subsidies, ergonomic workshops, and shared-use models—may help overcome current adoption barriers. With structured training and practical support, advanced magnification technologies are well-positioned to enhance clinical outcomes and precision in dentistry across Vietnam and similar contexts.

## Electronic supplementary material

Below is the link to the electronic supplementary material.

Supplementary Material 1

## Data Availability

Data is available upon reasonable request from the corresponding author.

## Abbreviations

3D-DOM: Three-dimensional digital operating microscope
CVI-S: Content Validity Index Score
DOM (conventional): Dental operating microscope
