# Supplementary material for: Utilization of magnification devices in Vietnam’s dental practice and education: an online survey
Source: BMC Oral Health. 2025 Jun 6;25:929. doi: 10.1186/s12903-025-06306-6 (PMC12142841; doi:10.1186/s12903-025-06306-6)
Supplement: Supplementary file 1 — Supplementary Material 1 [file 12903_2025_6306_MOESM1_ESM.pdf]

Section 1: Participant Information

1. Workplace (select all that apply)

- Specialized Dental Hospital
- Dentistry Department of a General Hospital
- Dental Training Institutions
- Private Dental Clinic
- Other (please specify)

2. Gender

- Male
- Female

3. Workplace Locations
(Please list all workplaces selected in Question 1)

4. Age

- <30 years old
- 30-35 years old
- 36-40 years old
- 41-45 years old
- 46-50 years old
- 50 years old

5. Educational Qualification (Select all that apply)

- Undergraduate Student
- Dentist
- Master's Degree
- Resident Doctor
- Specialist Level I
- Specialist Level II
- PhD
- Other (please specify)

6. Have you ever used or heard of the following magnification devices in dentistry? (Select all that apply)

- Loupes
- Operating Microscope
- 3D Microscope
- Never used or heard of
- Other (please specify)

7. How did you learn about the application of magnification devices in dentistry? (Select all that apply)

- Undergraduate training
- Postgraduate training
- Conferences/Workshops
- Colleagues/Friends
- Media/Social Networks
- Other (please specify)

8. In your opinion, which dental specialties can benefit from magnification devices? (Select all that apply)

- Disease diagnosis
- Implantology
- Tooth extractions
- Oral surgery
- Restorative dentistry
- Endodontics
- Fixed prosthodontics (including implant prosthetics)
- Removable prosthodontics (including implant prosthetics)
- Orthodontics
- Other (please specify)

9. Please assess your knowledge of magnification devices:

Level	Description
1	Little or no knowledge
2	Basic knowledge: Know basic features but not how to use/adjust
3	Moderate knowledge: Can use and adjust devices at a basic level
4	Good knowledge: Can use and fully adjust devices proficiently, fix minor issues
5	Expert knowledge: Understand operational principles, use devices fluently, and can fix most issues given the right tools

Self-assessment for each device:

- Loupes
- Operating Microscope
- 3D Microscope
- Other Magnification Devices

10. How did you learn to use magnification devices?

Device Type	No knowledge	Self-study	Peer-learning	Suppliers' experts	Universities' experts
Loupes					
Operating Microscope					
3D Microscope					
Other					

11. Have you ever used magnification devices in dentistry?

- Never used
- Have used before
- Other (please specify)

Sections 2-4: Experience with Specific Magnification Devices

Section 2:

12. Experience and Knowledge about Loupes

- Have you ever used loupes in dentistry? (Yes/No)

13. Years of experience using loupes

- <1 year
- 1-5 years
- 5-10 years
- 10 years

14. **Purpose of use** (Select all that apply)

- Teaching
- Research
- Diagnosis
- Treatment
- Other

15. Frequency of use for specific dental procedures
On a 5-point scale:
1: Never
2: Rarely (<2 times/week)
3: Occasionally (2-5 times/week)
4: Frequently (6-10 times/week)
5: Very frequently (>10 times/week)

- Diagnosis
- Implantology
- Tooth extraction
- Oral surgery
- Tooth fillings
- Endodontics
- Fixed restoration (including restorations on implants)
- Removable restorations
- Orthodontics
- Others

16. Use of Loupes in Endodontics

- Have you used loupes for: (select all that apply)
    - Canal orifice location
    - Managing broken instruments
    - Handling perforations/ledges
    - Obturation and filling placement

- Removing old filling materials

Section 3: Experience with Operating Microscopes

Experience and Knowledge about DOM

- Have you ever used DOM in dentistry? (Yes/No)

17. **Years of experience using DOM**
    - <1 year
    - 1-5 years
    - 5-10 years
    - 10 years

18. **Purpose of use** (Select all that apply)
    - Teaching
    - Research
    - Diagnosis
    - Treatment
    - Other

19. **Frequency of use for specific dental procedures**
    On a 5-point scale:
    1: Never
    2: Rarely (<2 times/week)
    3: Occasionally (2-5 times/week)
    4: Frequently (6-10 times/week)
    5: Very frequently (>10 times/week)
    - Diagnosis
    - Implantology
    - Tooth extraction
    - Oral surgery
    - Tooth fillings
    - Endodontics
    - Fixed restoration (including restorations on implants)
    - Removable restorations
    - Orthodontics
    - Others

20. Use of DOM in Endodontics

- Have you used DOM for: (select all that apply)

- Canal orifice location
- Managing broken instruments
- Handling perforations/ledges
- Obturation and filling placement
- Removing old filling materials

Section 4: Experience with 3D Microscopes

Experience and Knowledge about 3D DOM

- Have you ever used 3D DOM in dentistry? (Yes/No)

21. **Years of experience using 3D DOM**
    - <1 year
    - 1-5 years
    - 5-10 years
    - 10 years
22. **Purpose of use** (Select all that apply)
    - Teaching
    - Research
    - Diagnosis
    - Treatment
    - Other
23. **Frequency of use for specific dental procedures**
    On a 5-point scale:
    1: Never
    2: Rarely (<2 times/week)
    3: Occasionally (2-5 times/week)
    4: Frequently (6-10 times/week)
    5: Very frequently (>10 times/week)
    - Diagnosis
    - Implantology
    - Tooth extraction
    - Oral surgery
    - Tooth fillings
    - Endodontics
    - Fixed restoration (including restorations on implants)

- Removable restorations
- Orthodontics
- Others

24. Use of 3D DOM in Endodontics

- Have you used 3D DOM for: (select all that apply)
    - Canal orifice location
    - Managing broken instruments
    - Handling perforations/ledges
    - Obturation and filling placement
    - Removing old filling materials

Section 5: Accessibility and Effectiveness of Magnification Devices

25. Doctors are asked to rate statements on a scale from **0 (Strongly Disagree) to 5 (Strongly Agree)** regarding:

- Satisfaction with loupes/microscopes/3D microscopes
- Necessity of these devices in improving treatment quality

Evaluation of Magnification Devices in Different Specialties:

26. Participants rate **necessity** on a scale from 1 (Not Necessary) to 5 (Very Necessary) in different specialties:

- Diagnosis
- Implantology
- Tooth extraction
- Oral surgery
- Tooth fillings
- Endodontics
- Fixed restoration (including restorations on implants)
- Removable restorations
- Orthodontics
- Others

27. Limitations of Magnification Devices: (Check all that apply)

- High cost
- Heavy on the head
- Eye strain

- Uncomfortable posture
- Difficult to operate
- Blurred or double images
- Difficult maintenance
- Bulky design
- Prolonged treatment time
- Complicated to use
- No significant limitations

28. Advantages of Magnification Devices: (Check all that apply)

- Enhanced visualization
- Precision in operation
- Improved ergonomics
- Treatment with small instruments
- Reasonable cost
- Infection control
- No significant advantages

29. Future Plans for Using Magnification Devices (Check all that apply)

- Loupes
- Operating Microscope
- 3D Microscope
- No intention to use
